# Contemporary public image of the nursing profession in Saudi Arabia

**DOI:** 10.1186/s12912-020-00442-w

**Published:** 2020-06-09

**Authors:** Hala Elmorshedy, Abrar AlAmrani, Mona Hassan Ahmed Hassan, Amel Fayed, Susan Ann Albrecht

**Affiliations:** 1 College of Medicine, Princess Nourah Bint Abdulrahman University, P.O. Box: 84424, Riyadh, 1167 Saudi Arabia; 2 High Institute of Public Health, Alexandria University, Alexandria, Egypt; 3 School of Nursing and Midwifery, Queen’s University Belfast, Belfast, UK; 4 King Fahd Medical City, Riyadh, Saudi Arabia; 5 School of Nursing, University of Pittsburgh, Pittsburgh, USA

**Keywords:** Nursing profession, Socio-cultural barriers, Perception, Awareness, Saudi Arabia

## Abstract

**Background:**

In the Kingdom of Saudi Arabia, the nursing profession faces significant challenges including; failure to recruit high school students into nursing education, poor nursing identity, and lack of awareness about the nursing profession. The level of community awareness and public image of the nursing profession are critical to recruit and retain students into nursing education, and to improve nurses’ sense of identity.

**Aim:**

To explore the level of community awareness and public image of the nursing profession in Saudi Arabia.

**Methods:**

We conducted a cross-sectional study with a convenient sample of 502 adults including106 males and 396 females, their mean age was 22.93 ± 6.76 years. Data collected included; socio-cultural data, gender preference in getting nursing care, awareness, and perceived socio-cultural barriers to pursue a nursing career. Data were analyzed using SPSS version 21.0.

**Results:**

Only 32.5% preferred to get nursing care by Saudis. The nursing profession was not viewed as a respected job as 71.5% of participants would be ashamed of having a nurse in their families. The study revealed a low median knowledge score (50.0, IQR: 50.0–66.7)). The study highlighted a number of socio-cultural barriers to pursue a nursing career including; the gender-mixed working environment (35.9%), delayed marriage of female nurses (20.3%), and the negative effect of nursing profession on social life (64.5%).

**Conclusions:**

Half of the sample had a knowledge score below 50.0 out of 100. This level of poor awareness, in addition to socio-cultural perceived barriers are the main factors contributing to the negative public image of the nursing profession in Saudi Arabia. Understanding these factors could contribute to implementing focused intervention to improve the negative stereotype of the nursing profession among Saudis.

## Background

The shortage of nurses is a growing problem world-wide [1–4]. Many developed countries such as Australia, Canada, the UK, and the USA have undergone cycles of nursing shortages [5]. The problem is predicted to reach a crisis with negative consequences on the health care systems and patients’ safety [6, 7]. In view of the current situation of the nursing shortage, some countries have turned to international recruitment solutions. However, the international spatial movement of nursing labor may result in delivering inappropriate health care services. A wide variety of risky situations were attributed to the language barrier [8].

In Saudi Arabia, the first Bachelor of Science in Nursing (BSN) was established in 1976. Currently, there are 27 governmental universities, in addition to some other universities in the private sector offering BSN program; nevertheless, Saudisation of nursing profession is yet to be achieved. It is estimated that over 50% of the health care system is staffed by non-Saudis mainly recruited from India and the Philippine, with limited numbers of expatriate nurses are recruited from western countries, Malaysia and the Middle East [9–11]. This diversity creates language barriers which were perceived as a cause of workplace violence towards nurses as demonstrated in one study conducted in Riyadh [12]. In addition, language barriers may affect the quality of service offered to patients [13].

Saudisation of all the workforce sectors including the health care is one of the 2030 goals in the KSA- that is increasing the percentage of Saudi nationals participating in the workforce and reducing of the percentage of expatriates. Despite the efforts to establish this goal, the Saudi Ministry of Health statistics in 2012 revealed that expatriate staff represents 50% of the nursing workforce [9]. Of note, the current situation reflects a significant increase in the proportion of Saudis in the nursing profession compared to the previous report (Saudi Ministry of Health in 2008) which pointed out that Saudi nurses comprised only 29.1% of the total nursing workforce. However, the rate of Saudi nurses in private hospitals is as low as 4.1% [14, 15]. The underlying factors of shortage in Saudi nurses as addressed by Saudi educators and leaders include; cultural challenges, educational challenges, organizational challenges reflected by weak nursing authority and lack of acknowledgement for Saudi nurses and challenges in the working environment including; language barrier, gender-mixed working environment, and long working hours) [16].

Negative public perception of the nursing profession is a world-wide problem. Studies pointed out that improving the public image of nursing is fundamental in adopting a favorable nursing identity [17], while the poor image is likely to limit the consideration of nursing as a career choice [18, 19]. In the UK, one study pointed to: Students’ feelings of stress, not being valued, and having unmet expectations [20]. Of note in the KSA, most families might discourage their daughters to enroll in jobs with gender-mixed working environment [21].

Negative public image is influential for considering the nursing career as well as for retention of nurses in the nursing profession. Numerous studies highlighted the direct effect of the negative public image on a nurse’s sense of identity [10, 22–24]. A study in Saudi Arabia revealed that satisfaction of nurses is linked to a favorable nursing image [25]. In addition, the role of nursing education institutions is vital in shaping the nursing identity. Freshmen nursing students mainly reflect the general viewpoint of society as they only have a limited understanding about nursing [15, 26, 27]. A study conducted in Turkey demonstrated that students didn’t have sufficient knowledge regarding nursing at the beginning of their education, while an introduction to a nursing course has improved the students’ understanding of the nursing profession [27]. Also, media projection about nursing could play a fundamental role to influence the public image of nursing [22].

Most of the published studies in Saudi Arabia were focused on nursing students and graduates [10, 21, 24, 25], with very limited data about the public image which is crucial if missing in shaping the nursing identity in the KSA. Hence, the main objective of this study is to explore the level of community awareness and public image of the nursing profession in Saudi Arabia. Results would help to formulate intervention programs to improve the public image of the nursing profession leading to more recruitment, and retention of Saudis in the nursing profession.

## Methods

### Subjects

Adult Saudi individuals of both genders were invited to participate in the current study.

### Study settings

Princess Nuorah Bint Abdulrahman University (PNU) in Riyadh city, the capital of the KSA. To portray the majority of the Saudi community, we included students enrolled in non-health colleges only as they contributed to more than 90% of the total 50,000 students enrolled at PNU. Because PNU is a female university, and males in the Arab culture are influential in decision-making [28], we included adult male volunteers recruited from big malls in the city to our study sample. The variability in the socioeconomic status, age, and educational levels in mall visitors might better represent the community.

### Study design and sampling

We conducted a cross-sectional study using a convenient sample of 502 volunteers of both genders. A post-hoc power analysis revealed that a study of at least this size, assuming a prevalence of 50% negative perception, 7% allowable error in the estimate and 95% confidence, could detect differences with a power of 0.85.

### Ethical issues

The current study was conducted in accordance with the Declaration of Helsinki; the study protocol was approved by the Institutional Review Boards (IRB) of Princess Nourah Bint Abdulrahman University, Riyadh, KSA. Participants/guardians were consented verbally and were informed that their participation is voluntary, the data are confidential, and that they can withdraw from the study at any point. The IRB recommends the use of verbal consent in survey procedures unless information is obtained in such a manner that human subjects can be identified or subjects’ responses outside the research could place them at risk of criminal or civil liability or may damage their employability, or reputation.

### Tool of data collection

The researchers developed the questionnaire after a thorough review of the literature with modification to suit the Saudi culture. It included the following sections; 1- socio-cultural data such as age, gender, education, and if there are nurses among the family members. Sections 2–4 included items which could affect the public image about the nursing profession. The second section inquired about the preference of caregivers with regard to gender and nationality, rating of nursing skills offered by Saudi nurses, and stigma of the nursing profession. The third section was focused on awareness about nursing education and the nursing profession; it included six multiple response questions, the correct answer scored as one, and the wrong answer scored as zero. The total awareness score equaled 6. The fourth section addressed the perceived barriers to engage in a nursing career, and included seven questions structured on a Likert scales; the total perception score equaled 27. before application, the questionnaire was piloted on a sample of 20 individuals to determine the face validity, and necessary modifications were included. The final format was self-administered to participants volunteers.

### Analysis of data

Data were checked for completeness and were analyzed using SPSS version 21.0. Percentages, mean, median, and interquartile range (IQR) were used to describe the data. Awareness and perceived barrier scores were calculated as a percentage of the maximum score. As the distribution of scores was not symmetric, so it was presented as median with inter-quartile-range (IQR). Group comparison in median score was performed using the nonparametric Mann-Whitney test for two groups and the Kruskal Wallis test for more than two groups with Dunn post-hoc procedure in case of significant results and using the adjusted *p*-values to control type I error. A *p* value < 0.05 was considered significant. Multiple stepwise logistic regression analysis with Backward method was used to extract the most significant potential factors associated with awareness and perceived barriers of female nursing profession. Odds ratios of the outcomes adjusted for other variables was obtained with its 95% confidence interval.

## Results

The study encompassed 502 adult Saudis: 106 males (21.1%) and 396 females (78.9%), their mean age was 22.93 ± 6.76 with a median of 20 years. The majority were unmarried (80.3%). Overall, 74.3% were at university level of education or higher, out of the 106 males,70 (66.0%) were at university level of education or higher, while out of 396 females, 303 (76.5%) were at university education or higher (Table 1). In general, 81.5% preferred female nurses, while only 32.5% preferred Saudis to provide nursing care. Among those who experienced Saudi caregivers, only 40.8% rated the service as excellent. Less than half of male participants reported that they would marry a nurse, and as much as 71% of the total sample would be ashamed if they have a nurse in their family (Table 2).

**Table 1:** Distribution of the study sample according to socio-demographic characteristics

Sociodemographic characteristics, ***n*** = 502		Frequency	%
**Sex**	Male	106	21.1
	Female	396	78.9
**Age in years**	15-	166	33.1
	20-	223	44.4
	25+	113	22.5
	Mean ± SD	22.9 ± 6.8	
**Marital status**	Married	99	19.7
	Unmarried	403	80.3
**Education**	Below secondary	23	4.6
	Secondary (High school)	106	21.1
	* University or higher	373	74.3
**House**	Private	352	70.1
	Rented	150	29.9
**Female nurse in the family**	Yes	149	29.7
	No	353	70.3

*SD* Standard deviation

University or higher: Enrolled or completed their degrees

* Out of the 106 males,70 (66.0%) were at university level of education or higher, while out of 396 females, 303 (76.5%) were at university level of education or higher

**Table 2:** Preferences of nursing care and stigma of the nursing profession (*N* = 502)

Question	Response	Frequency	%
**Preference of care giver according to gender**	Male nurse	93	18.5
	Female nurse	409	81.5
**Preference of care giver according to nationality**	Saudi	163	32.5
	Non-Saudi	102	20.3
	Never mind	237	47.2
**Getting care by Saudi females**	Yes	299	59.6
	No	203	40.4
**Rating of Saudi female nursing care**^**a**^	Excellent	122	40.8
	Very good	112	37.5
	Good	55	18.4
	Bad	10	3.3
**Stigma of nursing** **(ashamed to have a nurse in my family)**	Strongly agree	246	49.0
	Agree	113	22.5
	Neutral	84	16.7
	Disagree	36	7.2
	Strongly disagree	23	4.6
**Preference to marry a female nurse**^**b**^	Yes	46	43.4
	No	60	56.6

^a^Asked to those who received care by Saudi female nurses (299) ^b^Asked to males only

Regarding awareness about the nursing profession, Table 3 shows that 61% of participants considered nursing as subordinate to physicians. Moreover, as much as 68.9% were unaware that nurses could achieve higher managerial positions, and about one-third of participants were unaware about the title of graduates from nursing collegiate programs. Additionally, participants demonstrated lack of knowledge concerning the length of time required to acquire a bachelor degree in nursing. In total, the median awareness score was only 50.0%, meaning that, half of participants scored less than 50.0% of the total score of 100%.

**Table 3:** Awareness about the nursing profession (*N* = 502)

Knowledge Item		Frequency	%
**Nursing duties**	Provide medical care, follow-up and health education	196	39.0
	Complementary to physician	306	61.0
**Job title of graduates of nursing programs below bachelor**	Nurse specialist	67	13.3
	Technical nurse	249	49.6
	Don’t know	186	37.1
**Job title of graduates of bachelor program**	Nurse specialist	286	57.0
	Technical nurse	52	10.4
	Don’t know	164	32.7
**Duration of bachelor’s degree (Including internship)**	Two years	22	4.4
	Three	54	10.8
	Four	97	19.3
	Five	152	30.3
	Don’t know	177	35.3
**Nursing has several specialties**	Yes	387	77.1
	No	115	22.9
**A nurse can be promoted to a hospital manager**	Yes	156	31.1
	No	346	68.9
^**a**^**Median score as percentage of the total score, (IQR)**		**50.0, (50.0–66.7)**	

*IQR* Inter-quartile-range (Q1 – Q3)

^a^ Score range 0–6 (0–100%), higher score indicates better knowledge

The median score of perceived barriers of the nursing profession was 51.85% of the maximum score. Differences in Language, and culture were perceived as barriers to get engaged in the nursing profession by 12% and 31.7% respectively. More than one-third of participants reported that gender-mixed working environment is a barrier for nursing practice, and about one-fifth consider that being a female nurse will delay marriage. In addition, 64.5% of participants claim that nursing profession affects female social life negatively (Table 4).

**Table 4:** Perceived barriers of public to engagement in the nursing profession, *n* = 502

Perceived barriers	Strongly agree		Agree		Neutral		Disagree		Strongly disagree	
	Freq.	%	Freq.	%	Freq.	%	Freq.	%	Freq.	%
Communication with different language	9	1.8	51	10.2	100	19.9	148	29.5	194	38.6
Communication with different culture	45	9.0	114	22.7	121	24.1	109	21.7	113	22.5
Being Saudi is viewed as a skilled nurse^a^	9	1.8	32	6.4	179	35.7	150	29.9	132	26.3
Poor public image of nursing profession	43	8.6	54	10.8	74	14.7	161	32.1	170	33.9
Mixed working environment	60	12.0	120	23.9	55	11.0	118	23.5	149	29.7
Delayed marriage of a female nurse	35	7.0	67	13.3	107	21.3	150	29.9	143	28.5
Nursing profession affects female social life	**Extremely**		**Moderately**			**Marginally**			**Not at all**	
	84	16.7	240	47.8		128	25.5		50	10.0
^**b**^**Median score as percentage of the total, (IQR)**	**51.85, (44.44–59.26)**									

*Freq* Frequency

*IQR* Interquartile range (Q1 -Q3) ^a^Reverse score order ^b^Score range 0–27 (0–100%)

Table 5 demonstrates factors associated with awareness and perceived barriers regarding the nursing profession. Higher level of education, having a nurse in the family, and marriage are associated with a higher awareness score. The perceived barrier score was higher among more educated individuals: 55.6, 51.9, and 48.2% for graduates of high school, university versus those who just can read and write respectively. Of note, the perceived barrier score was higher among those having a nurse in the family.

**Table 5:** Association between sociodemographic characteristics, awareness and perceived barriers of the nursing profession (*N* = 502)

Sample characteristics		Awareness		Perceived barriers	
		Median (IQR)	P^a^	Median (IQR)	P^a^
**Gender**	Male	**66.7** (50.0–83.3)	0.126	**51.9** (44.4–59.3)	0.863
	Female	**50.0** (50.0–66.7)		**51.9** (44.4–59.3)	
**Age in years**	15-	**50.0** (33.3–66.7)	0.069	**51.9** (44.4–59.3)	0.881
	20-	**50.0** (50.0–66.7)		**51.9** (44.4–59.3)	
	25+	**66.7** (50.0–83.3)		**51.9** (44.4–59.3)	
**Marital status**	Married	**66.7** (50.0–83.3)	0.001^b^	**51.9** (44.4–59.3)	0.225
	Unmarried	**50.0** (33.3–66.7)		**51.9** (44.4–59.3)	
**Education**	Read & write	**50.0** (33.3–66.7)	0.001^bc^	**48.2** (48.2–59.3)	0.043^bd^
	High school	**50.0** (33.3–66.7)		**55.6** (48.2–63.0)	
	University and higher	**66.7** (50.0–66.7)		**51.9** (44.4–59.3)	
**House**	Private	**50.0** (50.0–66.7)	0.541	**51.9** (44.4–59.3)	0.704
	Rented	**66.7** (33.3–66.7)		**51.9** (44.4–59.3)	
**Female nurse in the family**	Yes	**66.7** (50.0–66.7)	0.046^b^	**55.6** (48.2–63.0)	0.032^b^
	No	**50.0** (33.3–66.7)		**51.9** (44.4–59.3)	

^a^ Mann-Whitney test for two groups and Kruskal-Wallis test for more than two groups

^b^*P* < 0.05 (Significant), *IQR* Interquartile range (Q1 -Q3)

^c^ Post-hoc test indicated significant difference between University & higher versus Read & write

^d^ Post-hoc test indicated significant difference between University & higher versus High school

Table 6 shows that the unmarried participants and those with lower educational levels were mor likely to have poor knowledge. Those without a nurse family member had lower perceived barriers of nursing profession (AOR = 0.661, 95% CI: 0.443–0.985) while those who just read and write had higher perceived barriers (AOR = 1.679, 95% CI:1.062–2.655) compared to the highly educated respondents.

**Table 6:** Multiple stepwise logistic regression analysis of possible factors in relation to awareness and perceived barriers of the nursing profession (*N* = 502)

Sample characteristics		Awareness	Perceived barriers
		AOR 95% CI	AOR 95% CI
**Marital status**	Married	1	
	Unmarried	1.99 1.10–3.61	
**Education**	Read & write	2.74 1.15–6.51	1.68 1.062–2.66
	High school	1.81 1.12–2.92	
	University and higher	1	1
**Female nurse in the family**	Yes		1
	No		0.66 0.44–0.99

Dependent variables; Perceived barriers (≤ median = 0, >median = 1), Knowledge (Below median = 1, Median or above =0)

*AOR* Adjusted odds ratio, *CI* Confidence interval

## Discussion

The study revealed poor level of awareness about the nursing profession and distrust in Saudi caregivers. Surprisingly, about one-third only preferred to get the nursing care by Saudis. Moreover, the rating of Saudi caregivers was deemed excellent by only 40%. In addition, nearly three-quarters would be ashamed if they have a nurse in the family, and less than 50% of males prefer to marry a nurse. The study highlighted numerous barriers including; mixed working environment, delayed marriage of female nurses, and the negative effect of the nursing profession on social life. Regression analysis revealed no gender difference in the level of awareness, while those with low level of education, and un-married participants were more likely to have poor knowledge. Perceived barriers are more likely among less educated, while it was less likely among those reported that there were no nurses among their family members.

The low preference for Saudi caregivers might reflect poor image and distrust in the national caregivers; this would be very distressing and de-motivating for Saudi nurses and for those who might consider a nursing career in the future. In addition, nearly three-quarters of participant perceived that they would be ashamed of having a nurse among their families. In accordance with our results, a Study in Saudi Arabia revealed that nursing as a childhood dream was not appealing, nor family support was influential to pursue a nursing career [21]. One study reported that as much as 87.8% of Saudis didn’t prefer a nursing career for their family members [29]. Surprisingly, a Chinese study demonstrated that parents would not encourage their children to enroll in nursing education [30]. The observed positive perception of Omani university students about the nursing profession might reflect a reporting bias, since the numbers of the nursing students outweigh those from other disciplines [31]. Secondly, other factors concerning the heath care policies to recruit nationals and media marketing of the nursing profession in Oman should not be ignored. Of note, underestimating the skills of Saudi nurses should gain more attention by Saudi leaders and educators in the health care sectors as it might reflect a real gap between theory and practice as highlighted in a recent study [16].

However, in this study, the preference of females as caregivers stems from the perception that nursing is women’s occupation. In addition, cultural norms reject getting care by opposite gender especially for females.

Regarding the level of awareness about the nursing profession, it is striking that most of participants in the present study viewed the role of nursing in health care settings as complementary to physicians, and about one-third do not know the exact job title of graduates from nursing college or institutes. Additionally, participants don’t know even the duration required to get a bachelor’s degree in nursing.

The level of public awareness about the nursing profession is critical for how they view the nursing career. Previous studies across different cultures confirmed the perceived attenuated role of nurses compared to physicians. These negative stereotypes view nurses as having no status and describe them as physicians’ assistants and ancillary workers [26, 30, 32]. In a study conducted in Iran, students emphasized that the social status of nurses is not desirable, as they are considered handmaidens of physicians and dutiful hospital employees. For example, one student mentioned: “although nurses are sometimes perceived as educated, their main task is to obey and follow physicians’ orders and their caring skills are not appreciated” [22].

Similarly, a recent study in Saudi Arabia which included over 700 participants of both genders, revealed that more than one-third of participants perceived nursing as a profession that is auxiliary and subservient to physicians, and therefore study participants claim that there is no need for higher academic qualifications [21]. This means that the community in Saudi Arabia doesn’t value the skills and competencies acquired through higher academic education.

Despite the sincere efforts of Saudisation of the workforce in the KSA, expatriates remain to constitute a considerable proportion of the nursing workforce [25]. Saudi culture may regard the nursing profession unfavorable work atmosphere and hence discourage their children to pursue a nursing career. The present study revealed that a considerable proportion of the study sample amounted to 64.5% claimed negative consequences of the nursing profession on social life because of long working hours and night shifts, other barriers include delayed marriage and gender-mixed working environment. Regression analysis showed that less educated individuals were more likely to perceive barriers, probably because of lack of knowledge. The observation that barriers were more likely if there were nurses among the family further indicates the challenges faced by Saudi nurses.

It is likely that the global image of the nursing profession is negative. Previous studies demonstrated that nursing is viewed as a less desirable profession because of difficult working conditions, inadequate financial compensation, low level of autonomy, limited career opportunities, and being viewed as ancillary members of the healthcare team [33].

Finally, this study demonstrates low level of public awareness and reflects poor public image of the nursing profession. Such information is paramount for effective interventions to raise community awareness and modify the negative image of the nursing profession. Hopefully, the nursing identity will be improved leading to recruitment of high achievers and retention of candidates into the nursing career.

### Limitations of the study

The study was conducted in Riyadh the capital city of the KSA, so generalizability of the study is limited to similar settings. Should we consider sub-urban and rural communities, the data collection tool needs further validation and reliability testing. The convenient sampling technique, and the unbalanced gender representation add to study limitations.

## Conclusions

The study demonstrated a negative public image of the nursing profession, only, one-third of participants preferred to get nursing care by Saudis, and the nursing profession was not viewed as a respected job by majority. Awareness about the nursing profession was poor, as half of participants scored below 50% out of 100. The study highlighted a number of barriers to pursue a nursing career including; working in a gender-mixed environment, delayed marriage of females, and poor social life. These findings are crucial to implementing focused intervention strategies in the future to improve the public perception of the nursing profession in the KSA.

## Supplementary information

**Additional file 1.**

## Data Availability

The datasets of the current study are available from the corresponding author on reasonable request.

## Abbreviations

BSN: Bachelor of Science in Nursing
KSA: Kingdom of Saudi Arabia
UK: United Kingdom
PNU: Princess Nourah Bint Abdulrahman University
IQR: Interquartile range
AOR: Adjusted odds ratio
CI: Confidence interval
