# Supplementary material for: Contemporary public image of the nursing profession in Saudi Arabia
Source: BMC Nurs. 2020 Jun 9;19:47. doi: 10.1186/s12912-020-00442-w (PMC7285542; doi:10.1186/s12912-020-00442-w)
Supplement: Supplementary file 1 — Additional file 1. [file 12912_2020_442_MOESM1_ESM.docx]

**Measuring public image, knowledge & barriers of the nursing profession in Saudi Arabia**

You are being asked to voluntarily participate in this survey research study “Measuring public image, knowledge & barriers of the nursing profession in Saudi Arabia”

If you agree to participate, your participation will involve completing a survey. It should take no more than 10 minutes. You may choose not to answer some or all of the questions. Your name will not appear on your completed survey, and no identifying information is being collected as part of this survey.

Only the study team will have access to the information that you provide, which will remain anonymous. Data from all respondents will be summarized in reports.

You can obtain further information from the principal investigator, Dr. Hala Elmorshedy. If you have questions concerning your rights as a research subject, you may call the PNU Institutional Review Board office at 288-9999 ext. 26913.

Completing this survey indicates your voluntary agreement to participate. By participating in the survey, you are giving permission for the investigator to use your information for research purposes.

Thank you.

Dr. Hala Elmorshedy

[elmorshedyh@hotmail.com](mailto:elmorshedyh@hotmail.com)

| **Questionnaire: Measuring public image, knowledge & barriers of the nursing profession in Saudi Arabia** | | | | | | | | | | | | | | | | | | | | | | | | | |  |  |  |  |
| --- | --- | --- | --- | --- | --- | --- | --- | --- | --- | --- | --- | --- | --- | --- | --- | --- | --- | --- | --- | --- | --- | --- | --- | --- | --- | --- | --- | --- | --- |
|  | | | | | | | | | | | | | | | | | | | | | | | | | |  |  |  |  |
| **Socio-cultural Data: Answer questions from 1-6 by ticking the answer that represents you** | | | | | | | | | | | | | | | | | | | | | | | | | |  |  |  |  |
| **1.Gender** | | | | | - Male | - Female | | | | |  | | | |  | | | | |  | | | | | |  |  |  |  |
| **2. Write your age** | | | | | ………………………… |  | | | |  | |  | | | |  | | | | | | |  | | | | |  |  |
| **3. Marital status** | | | | | - Single | - Married | | | |  | |  | | | |  | | | | | | |  | | | | |  |  |
| **4. Educational level** | | | | - Illiterate - Completed secondary school | | | | - Read & write - University degree | | | | | | | | - Completed primary school - Above university | | | | | | | | | | |  |  |  |
| **5. Monthly Income in SR** | | | | | - < 5,000 | - 5,000-10,000 | | | | - 10,000 - 14,000 | | | | | | | | - ≥ 15,000 | | | | | | | |  |  |  |  |
| **6. Housing** | | - Owned a house | | | | - Rented house | | | | | | | | | |  |  | | | | | | |  | | | | |  |
| **Knowledge about nursing profession: Answer questions from 7-13 by ticking the answer that represents you** | | | | | | | | | | | | | | | | | | | |  | | | | |  |  |  |  |  |
| **7. Role of nurses in medical care include which one of the followings: *(tick all that apply)*** | | | | | | - Patients’ cleaning care - Giving injections and medications - Complementary to physicians | | | | | | | | | | | - Monitoring and follow-up - Health education | | | | | | | |  |  |  |  |  |
| **8. Title of graduates of non-university nursing programs** | | | | | | - Nursing specialist | | | | | | | - Nursing technician | | | | | | | | - I don’t know | | | |  |  |  |  |  |
| **9. Title of graduates of university nursing programs** | | | | | | - Nursing specialist | | | | | | | - Nursing technician | | | | | | | | - I don’t know | | | |  |  |  |  |  |
| **10. Years of BSN program** | | | | | | - 2 years | | | | | | - 4 years | | | - 5 years | | | | | | - I don’t know | | | |  |  |  |  |  |
| **11. Nursing have several specialties** | | | | | | - Yes | | | | | | - No | | | | | | | | | | | | |  |  |  |  |  |
| **12. Nurses can be promoted to managerial positions** | | | | | | - Yes | | | | | | - No | | | | | | | | | | | | |  |  |  |  |  |
| **13 I have a nurse in my family** | | | | | | - Yes | | | | | | - No | | | | | | | | | | | | |  |  |  |  |  |
|  | |  | | | | |  |  | | | | | | | | | |  | | | | | |  | | | |  |  |

| **Barriers of nursing profession: Answer questions from 14-20 by ticking the answer that represents you** | | | | | | | |
| --- | --- | --- | --- | --- | --- | --- | --- |
|  | Strongly agree | Agree | Don’t know | | Disagree | | Strongly disagree |
| **14. Poor public image of the nursing profession** |  |  |  | |  | |  |
| **15. Gender-mixed environment** |  |  |  | |  | |  |
| **16. Communication with different language** |  |  |  | |  | |  |
| **17. Communication with different cultures** |  |  |  | |  | |  |
| **18. Saudi nurses are underestimated** |  |  |  | |  | |  |
| **19. Delayed marriage of females working in the nursing profession** |  |  |  | |  | |  |
| **20. Nursing profession affects female social life negatively** | - extremely | - Moderately | | - Marginally | | - Not at all | |

| **Opinion of nursing profession: Answer questions from 21-26 by ticking the answer that represents you** | | | | | | | | | |  |  |
| --- | --- | --- | --- | --- | --- | --- | --- | --- | --- | --- | --- |
| **21. Preference of getting nursing care** | - A female nurse | | | | - A male nurse | | | | |  |  |
| **22. preference of nationality to provide nursing care** | - Saudis | | - Non-Saudis | | | | - Nationality doesn’t matter | | |  |  |
| **23. Did you get nursing care to you or any of your family members by Saudi nurses?** | - Yes | | - No | | | | | | |  |  |
| **24. How do you rate the skills of Saudi nurses?**  **(Asked to those received nursing care by Saudis)** | - Excellent | | - Very Good | | | - Good | | - Bad | | |  |
| **25. Do you accept to marry a Saudi nurse?**  **(Asked to males)** | - Yes | | - No | | | | | | |  |  |
| **26. I feel ashamed if I have a nurse in my family** | - Strongly agree | - Agree | | - Don’t know | | - Disagree | | | - Strongly disagree | | |
